# Pulsed field ablation of ventricular arrhythmias arising from intracavitary structures: insights from a clinical case series

**DOI:** 10.1007/s10840-025-02072-1

**Published:** 2025-06-02

**Authors:** Kasun De Silva, Tai Chung So, Samual Turnbull, Max Bickley, Kenji Hashimoto, Ashwin Bhaskaran, Saurabh Kumar

**Affiliations:** 1 Department of Cardiology, Westmead Hospital, Corner Hawkesbury, and Darcy Roads, Sydney, NSW 2145 Australia; 2 Westmead Applied Research Centre, University of Sydney, Sydney, NSW Australia

**Keywords:** Pulsed field ablation, Premature ventricular complex, Catheter ablation, Ventricular arrhythmia

## Abstract

**Introduction:**

Premature ventricular complexes (PVCs) from intracavitary structures, such as papillary muscles and the moderator band, can be challenging to treat. Pulsed field ablation (PFA) offers a novel strategy for treating these arrhythmias.

**Methods:**

Between 2023 and 2024, three patients with intracavitary PVCs (two with PVC-mediated ventricular fibrillation) underwent PFA at a tertiary referral centre. Electroanatomic mapping was performed and, with intracardiac echocardiography (ICE) guidance, PFA was delivered using a pentaspline Farapulse catheter, with adjuvant radiofrequency (RF) ablation as needed.

**Results:**

All patients had successful abolition of PVCs. PFA delivery was feasible and safe, with excellent success despite prior RF ablation failures though one patient required adjuvant RF ablation. The only complication was a persistent right bundle branch block (RBBB) after PFA delivery to the moderator band. Follow-up showed significant reductions in PVC burden and no further ventricular fibrillation (VF) episodes. The mean procedural duration was 153.67 ± 31.71 min, and the mean fluoroscopy time was 14.38 ± 6.74 min.

**Conclusion:**

This is a preliminary proof of concept report for ablation of PVCs from intracavitary structures, warranting further validation studies.

## Introduction

Premature ventricular complexes (PVCs) can arise from complex intracavitary mobile structures including left and right ventricular papillary muscles and the right ventricular moderator band. PVCs from these sites, though forming only 2–5% of all ablation cases [1, 2] can be extremely challenging to treat. Long-term arrhythmia-free survival rates can be as low as 60% [1, 3] for papillary muscle PVCs and 80% for PVCs arising from the moderator band [4], often with the requirement for multiple procedures and prolonged procedural times [2, 5]. Challenges include anatomical variations, including differences in the size and position of these structures [3] radiofrequency (RF)-induced automaticity [4], deep intramural arrhythmia origins in some cases, and, most importantly, the shape and mobile nature of these structures, which can result in poor catheter contact and stability during lesion delivery [3]. To address the latter, technologies beyond traditional RF ablation have been shown to have promise, including focal and balloon cryoablation [2, 6, 8]. Although these technologies have improved procedural success, ablation is still challenging with procedural duration in a recent series exceeding 4 h [2].

Pulsed field ablation (PFA) is an emerging technology designed to cause irreversible electroporation of cell membranes, leading to cell death and lesion formation in both healthy and diseased myocardium [9, 10]. Whilst the depth of lesion formation is still dependant on catheter contact in PFA [10], a recent pre-clinical study using a large footprint lattice-tip catheter showed good lesion formation in papillary muscles and the moderator band in a swine model with preservation of valvular function [11]. Whilst designed to target cardiomyocytes, PFA has also been shown to affect the electrical conduction system and eliminate Purkinje fibre potentials, which can reduce vulnerability to ventricular fibrillation (VF) [12]. Given the dense Purkinje fibre network and propensity for PVC-mediated VF from these structures, we hypothesised that PFA would be a useful strategy to target PVCs from these intracavitary structures. Furthermore, it is conceivable that such a large footprint system may overcome limits of contact and stability and RF-induced automaticity which could improve outcomes in these cases.

Here we present a proof-of-concept case series of ventricular arrhythmia ablation of the intracavity structures—namely, the moderator band and right and left ventricular papillary muscle—using PFA.

## Methods

Between 2023 and 2024, three patients underwent PFA for treatment of intracavitary PVCs at a single tertiary referral centre (Westmead Hospital) and were included in this case series. All patients provided informed consent to the off-label use of PFA for targeting their arrhythmia prior to their procedure. Data for this study was collected as a part of the VA-West registry, approved by the Western Sydney Local Health District Human Research Ethics Committee.

Transthoracic echocardiography ± cardiac multi-detector computed tomography (CT) ± cardiac magnetic resonance imaging was obtained on patients during pre-procedural workup to assess for structural heart disease and evaluate ventricular function. Holter monitoring for a minimum of 24 h was conducted to evaluate the pre-procedural PVC burden.

### Electroanatomic mapping

Our approach to mapping PVCs has been described previously [13]. Antiarrhythmic drugs (AADs) were ceased at least five half-lives prior to the procedure, except in the case of an emergent indication. General anaesthesia was administered after recording the 12-lead electrocardiogram pattern of the clinical PVC.

Vascular access was obtained under ultrasound guidance. A series of venous sheathes were inserted: (1) an SL3 sheath (Abbott Medical, IL) to perform coronary sinus venography and insert a decapolar catheter into the coronary sinus; (2) a short sheath for the insertion of a quadripolar catheter into the right ventricular apex; (3) a short sheath for an ICE catheter (CARTO; Biosense Webster, CA); and finally, (4) for the LV, an SL0 sheath (Abbott Medical, IL) for transeptal puncture under ICE guidance, exchanged for a large curved Agilis catheter (Abbott Medical, IL) for mapping, or, for RV; a small curved Agilis catheter for mapping and ablation of the right ventricle. These were subsequently exchanged for the Faradrive steerable sheath (Boston Scientific, MA) for PFA.

ICE was used with CartoSound (Biosense Webster, CA) to create endocardial shells of the chamber of interest, with particular attention given to demarcating intracavitary structures. It was also utilised to monitor catheter contact of the activation mapping (DecaNav, Biosense Webster, CA), RF ablation and PFA catheter.

If PVCs were too scarce to permit activation mapping, several provocation manoeuvres were employed. Burst RV pacing down to ventricular refractoriness was performed from the RV apex. This was then repeated on the highest tolerated dose of isoprenaline (up to 40 µG/min). Programmed electrical stimulation was also performed using a 400-ms drive train with 4 extrastimuli beginning at 300 ms, decrementing by 10 ms down to ventricular refractoriness. Sustained VT was defined as monomorphic VA with duration > 10 s. Activation mapping was performed to find site of earliest origin (and position on intracavitary structures was confirmed by ICE.

If there were paucity of PVCs to permit activation mapping even with provocation, bipolar pacing from the ablation catheter at a fixed rate of 600 ms (or 10 ms below baseline rate) with an output of 10 mA and 2 ms pulse width was performed. Tissue contact was monitored aiming for contact force (CF) of ≥ 10 g. If there was no pace capture, the pacing output was increased to 10 mA and 9 ms as required. Captured beats were analyzed using the PASO (Carto) algorithm.

### Radiofrequency and pulsed field ablation

For PFA, a 31-mm multielectrode pentaspline Farapulse (Boston Scientific, MA) PFA catheter was advanced into RV or LV via a deflectable Faradrive steerable sheath. It was advanced into the ventricle over the wire in basket configuration. The catheter was visualised at all times by ICE entering into the ventricle to monitor for entanglement with valvular chordae. The manipulation of the PFA catheter was guided by ICE. The catheter was predominantly used in ‘basket’ configuration; however, the ‘flower’ configuration was attempted if contact was poor. It was visualised as a virtual catheter in CARTO 3D mapping system (connection of 3rd electrode of every spline to the CARTO system). The contact and stability of catheter with tissue was visualised under ICE. Repetitive applications of PFA using standard settings: 2.0-kV output, 5 biphasic and bipolar pulses 200 ms (ms) in duration each with 300 ms pause (2.5 s per application), aiming for suppression of PVCs. Importantly, prior to lesion delivery with PFA, the position and contact of the PFA catheter in either ‘basket’ or ‘flower’ configuration was confirmed in precise detail with ICE. This safety measure confirmed that PFA splines were not inadvertently in contact with and inadvertently ablating surrounding structures.

Where required, adjuvant RF ablation was performed using a 3.5-mm-tip open-irrigation catheter ThermoCool SmartTouch (Biosense Webster, CA). Ablation was delivered aiming for a contact force of ≥ 10 g. RF energy of up to 50 watts was delivered, aiming for an impedance drop of between 10 and 20 ohms. As with PFA, catheter position and stability were determined with ICE.

### Follow-up

All patients underwent a 24-h period of cardiac monitoring immediately following the procedure. After discharge, patients were followed up through a combination of outpatient clinical reviews, up to 5-day Holter monitoring and/or device checks to monitor ventricular arrhythmia and PVC counter. Hospital medical records and outpatient clinic assessments were used to complete clinical follow-up. Follow-up was defined as the time from final ablation procedure to the last documented clinical review.

## Results

The baseline characteristics of the three patients are summarised in Table 1. The procedural characteristics are summarised in Table 2.

**Table 1:** Patient characteristics

Case number	Age	Sex	LVEF (%)	Cardiomyopathy	IHD	VA	AADs	Previous ablations
1	55	Male	67	Possible right ventricular dysplasia on RV biopsy (fat infiltration and fibrosis) but negative gene panel and normal endocardial/epicardial voltage mapping	No	PVC induced VF	Pre-ablation: Metoprolol XL Failed/Not tolerated: Amiodarone, flecainide, carvedilol, verapamil, mexiletine Phenytoin	5
2	74	Female	56	Mild RV systolic dysfunction on CMR not meeting ARVC criteria. Negative gene panel	No	PVC	Pre-ablation: bisoprolol Failed/not tolerated: verapamil	1
3	69	Male	41	Post-infarction cardiomyopathy. CMR demonstrated subendocardial scar involving mid-distal anterior/anteroseptum and basal-mid inferior wall including base/body of posteromedial papillary muscle	Yes	PVC induced VF	Pre-ablation: sotalol Failed/not tolerated: amiodarone	0

*AADs* antiarrhythmic drugs, *ARVC* arrhythmogenic right ventricular cardiomyopathy, *CMR* cardiac magnetic resonance imaging, *IHD* ischaemic heart disease, *LVEF* left ventricular ejection fraction, *PVC* premature ventricular complex, *RV* right ventricle, *VF* ventricular fibrillation

**Table 2:** Procedural characteristics

Case number	PVC site	PVC description	Earliest activation (ms)	Best pace map score (%)	PFA applications	RFA touch up	RFA touch up applications	Procedural time (mins)	Fluoroscopy time (mins)
1	RV moderator band	PVC1: LB transition V6, LS axis PVC2: LB -ve concordance, Left axis (II/III discordance) PVC3: LB -ve concordance, LS axis	-	PVC1:93 PVC2: 88 PVC3: 83	28 (in basket formation)	No	-	197	12.22
2	RV anterior papillary muscle/moderator band complex	LB -ve concordance, RS axis	− 32	98	8 (in basket formation)	Yes	16	122	7.41
3	LV posteromedial papillary muscle	RB, transition V4, LS axis	− 26	-	30 (basket and flower)	No	-	142	23.5

*LB* left bundle branch block morphology, *LVEF* left ventricular ejection fraction, *LI* left inferior, *LS* left superior, *LV* left ventricle, *PFA* pulsed field ablation, *PVC* premature ventricular complex, *RB* right bundle branch block morphology, *RFA* radiofrequency ablation, *RV* right ventricle, *VF* ventricular fibrillation

### Case 1

A 55-year-old man, with history of PVC mediated VF arrest presented with recurrent implantable cardioverter defibrillator (ICD) shocks. His first episode of VF was at age 29, and at that stage, an ICD was implanted. An RV biopsy after initial presentation suggested possible right ventricular dysplasia (fatty infiltration and fibrosis in RV myocardium), and 12-lead ECGs showed frequent left bundle branch block morphology PVCs. However, gene testing revealed two variants of unknown significance that were non-phenotypical of arrhythmogenic right ventricular cardiomyopathy (ARVC). An endocardial/epicardial substrate map was within normal limits. He underwent four endocardial RF catheter ablations followed by one endocardial/epicardial RF catheter ablation, but PVC-mediated VF and shocks persisted. During his most recent procedure, the PVC was mapped to the moderator band, and RF energy was applied without success.

**Fig. 1:**
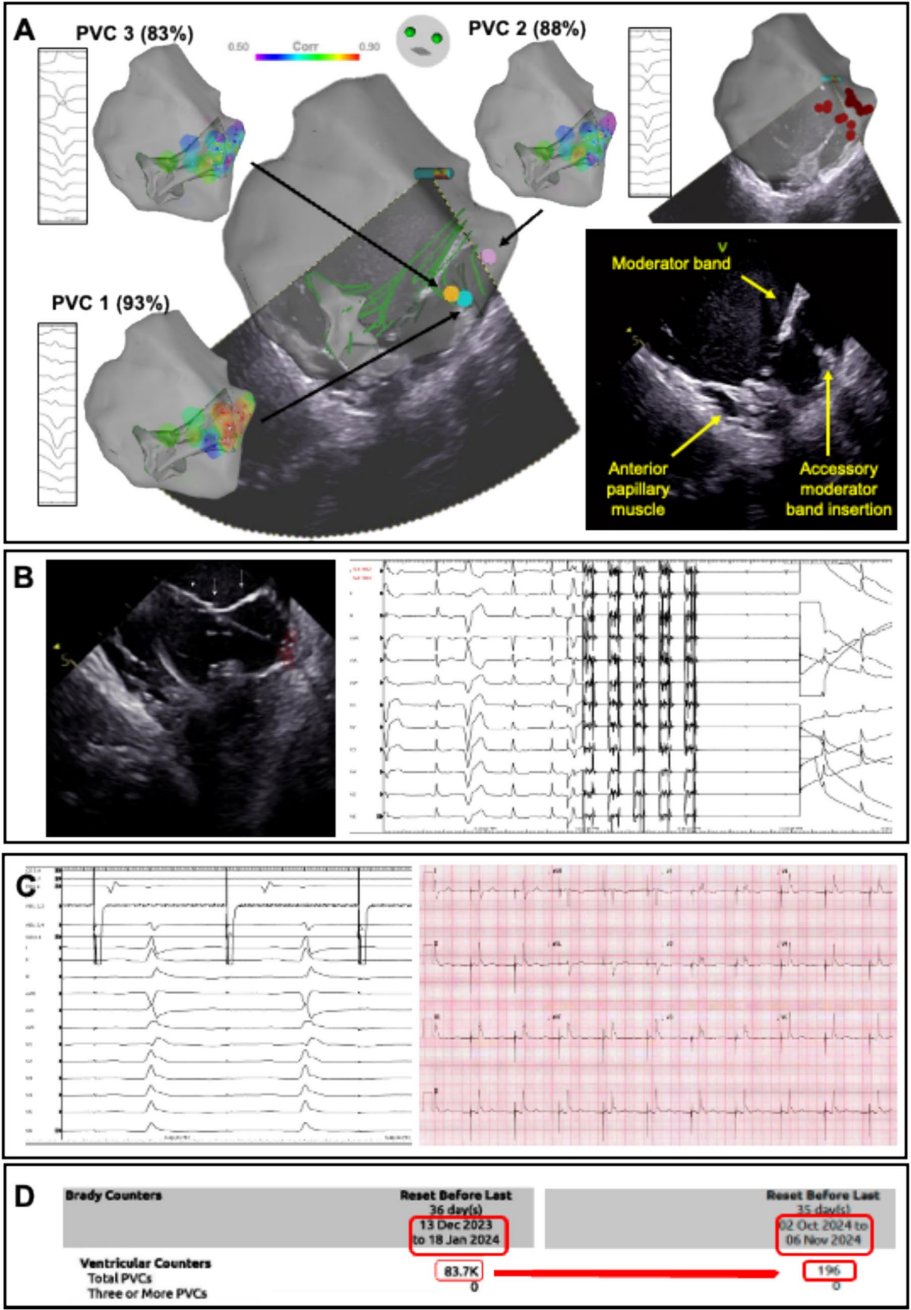
Case 1: **A** The morphology of the right ventricular moderator band; its insertion into the right ventricular wall and insertion into the right ventricular anterior papillary muscle is appreciable on intracardiac echocardiography (ICE). Three PVC morphologies were encountered during the case. Pace map scores superimposed upon the moderator band–right ventricular papillary muscle complex are shown. Best pace map score for PVC1 was 93% localised to an accessory insertion from the right ventricular moderator band to the right ventricular apex (blue dot). Best pace map score for PVC 2 was 88% to the moderator band body. Best pace map for PVC 1 was 83% to a similar region to PVC1. On the right most electroanatomic map, the location of radiofrequency lesions to target these sites is displayed. **B** ICE demonstrates good catheter contact of the Farapulse catheter in “basket” formation to the region of interest. A single application of PFA (2kv, 5 pulses, 200 ms duration for each packet with 300 ms gap) is demonstrated. **C** Post ablation testing for ventricular capture with the ablation catheter demonstrates electrical inexcitability with newly noted right bundle branch block (also seen on post procedural ECG). **D** Post PFA procedure, dramatic reduction in PVC counter on ICD follow-up (293 days post ablation)

In view of failure and/or intolerance of multiple anti-arrhythmic drugs, including amiodarone, a high PVC burden on Holter (10.13%) and PVC-triggered VF, repeat catheter ablation was offered. Three PVC morphologies were identified (summarised in Table 2); however, they were too infrequent for activation mapping, even with provocation. Pace mapping with ICE guidance showed good matches at anterior RV free wall at an accessory insertion site of moderator band into the RV wall (Fig. 1A). RF ablation at the best pace map area failed to eliminate the PVCs. In view of prior failed ablations, adjuvant PFA was employed.

The PFA catheter was advanced into RV, and its manipulation was guided by ICE. The catheter was in basket configuration and was visualised as a virtual lasso-shaped catheter on the electroanatomic mapping system. The contact and stability of catheter with tissue was visualised under ICE (Fig. 1). A total of 28 applications were performed in the previously ablated area and over the moderator band. There was loss of capture in PFA ablated area at high output pacing (20 mA/9 ms) (Fig. 1). The patient developed a right bundle branch block (RBBB) after PFA which persisted after the case. The ICD lead parameters remained stable after the procedure. There was no significant haemoglobin reduction post procedure. The PVC counter on his device decreased from 83,700 over a 36-day period prior to ablation to 196 (Fig. 1), with no further episodes of ventricular fibrillation observed during a follow-up period of 293 days after ablation.

### Case 2

A 74-year-old woman presented with symptomatic high-burden PVCs (35% on Holter) despite taking slow-release Verapamil 180 mg. Cardiac MRI showed a mildly dilated RV with reduced systolic function (RVEF 41%). However, she did not otherwise meet ARVC criteria. A genetic panel for cardiomyopathy demonstrated no medically significant variants. She underwent RF ablation for a moderator band PVC, during which the septal insertion of RV moderator band was ablated. Despite this, she had persistent symptoms and a high PVC burden (27%).

**Fig. 2:**
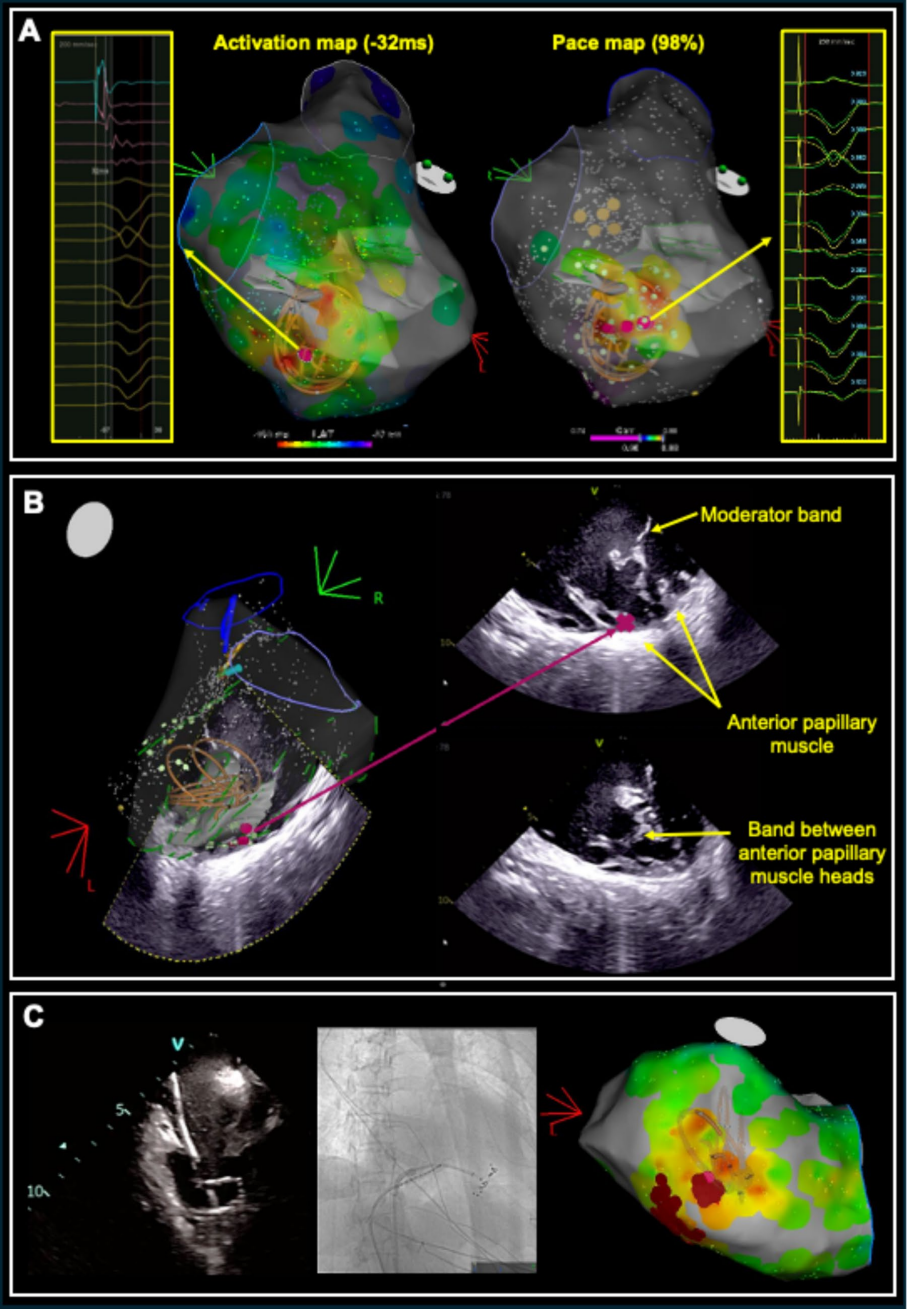
Case 2: **A** Activation and pace map demonstrates localisation of the PVC to the right ventricular papillary muscle (pink dot is best site). The map has been made transparent to demonstrate location of PFA catheter (shown on this electroanatomic map as a lasso-shaped virtual catheter). **B** Intracardiac echocardiography (ICE) demonstrates a complex right ventricular papillary muscle (band noted between two heads), localising the region to the base of the muscle. **C** ICE demonstrates manoeuvring of PFA catheter in “basket” formation into the RV apex. Fluoroscopy (right anterior oblique 30) demonstrates difficulty to advance catheter further to apex. Electroanatomic map demonstrates adjuvant lesion delivery with radiofrequency ablation

Repeat catheter ablation was offered. The PVC was left bundle branch block morphology, negative concordance and left superior axis. Activation map with ICE guidance showed earliest activation at apical lateral RV at the base of a complex RV anterior papillary muscle − 32 ms pre-QRS and with 98% pace match (Fig. 2). Due to prior failed ablation and the complexity of the RV moderator band-papillary muscle complex, PFA was employed. The PFA catheter was guided by fluoroscopy and ICE. A total of eight applications of PFA were performed at the site of best activation/pace map. However, the PFA catheter was not able to advance further apically to contact the apical side of the target area, which was still captured electrically when paced at high output (10 mA/9 ms). Therefore, adjuvant RF ablation was performed to the apical area where the PFA catheter could not reach. No PVCs were observed after the ablation, despite high-dose isoproterenol infusion (10 mcg/min). There was no significant reduction in haemoglobin or other complications. At 50 days follow-up, the PVC burden on a 5-day Holter monitor was reduced to 1.5%.

### Case 3

A 69-year-old man with history of post-infarction cardiomyopathy (LVEF 41%, moderate mitral regurgitation) presented with an ICD shock due to PVC-triggered VF 4 years ago. He was started on amiodarone but subsequently developed amiodarone-induced thyroiditis. Following the cessation of amiodarone and the commencement of sotalol, he continued to experience symptoms. Cardiac MRI demonstrated subendocardial scarring in the mid-distal anterior and anteroseptal wall and basal-mid inferior wall. Late gadolinium enhancement was also noted on the base and body of the posteromedial papillary muscle.

**Fig. 3:**
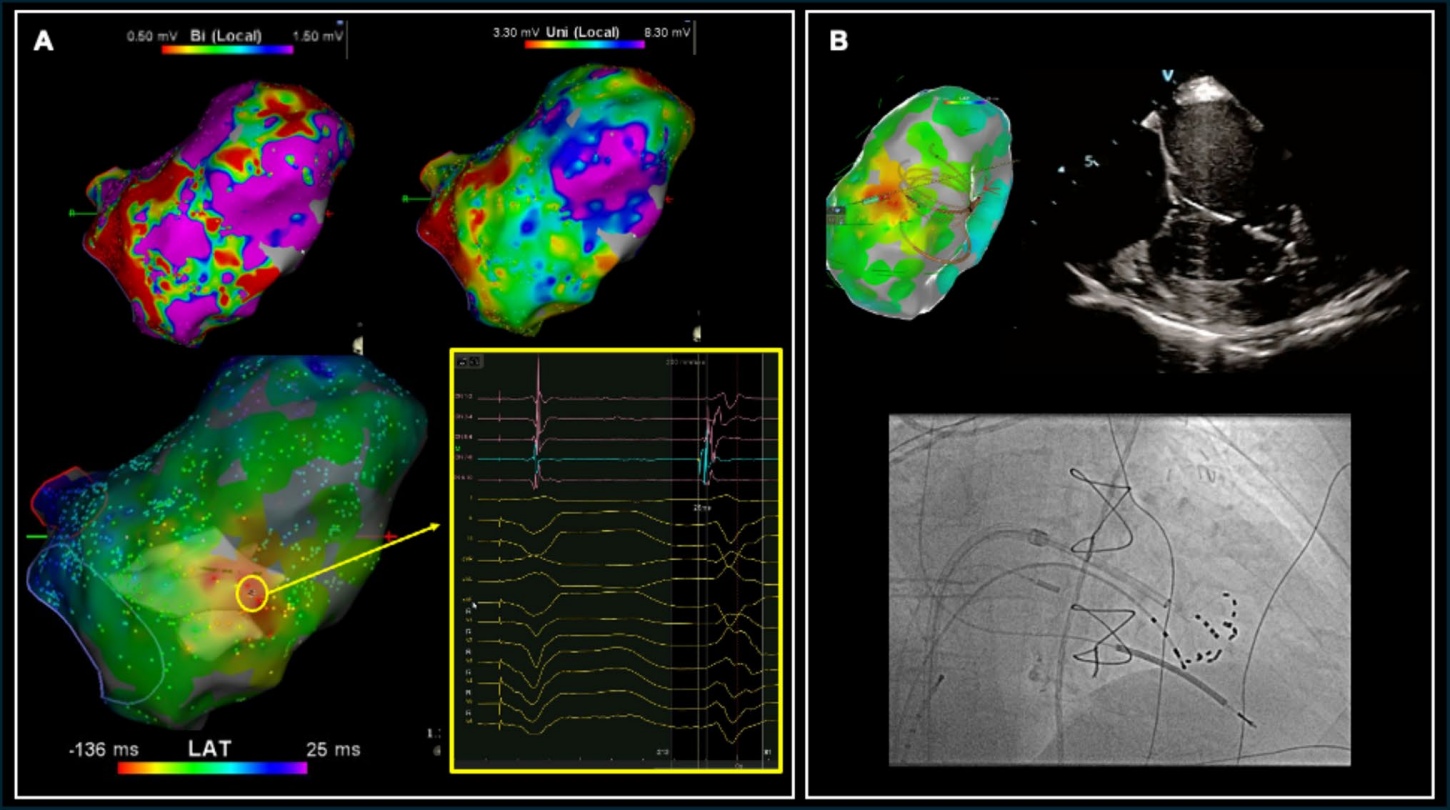
Case 3: **A** Bipolar and unipolar voltage maps demonstrate post-infarction scarring of the left ventricle. The activation map (made transparent to show the papillary muscles delineated by intracardiac echocardiography) shows earliest site of activation at the base of the posteromedial papillary muscle. The site of earliest activation − 26 ms pre QRS shows a multicomponent fractionated electrogram without clear isolated Purkinje potential at the base of the posteromedial papillary muscle. **B** Electroanatomic map shows the location of the PFA catheter during lesion delivery, also shown on intracardiac echocardiography where the catheter can be seen with good contact with the papillary muscles. The fluoroscopy image shows flexion of the catheter to aid contact with the posteromedial papillary muscle

Catheter ablation was offered due to failed medical treatment. Substrate mapping demonstrated a low voltage area over basal to middle inferior wall and apical region. The PVC was a right bundle branch block morphology with transition in V4 and left superior axis. Activation mapping of the PVC showed a focal centrifugal activation pattern with earliest activation time − 26 ms pre QRS at the base of the posteromedial papillary muscle (Fig. 3). The local electrograms at this site were fractionated. Due to the known scarring in the posteromedial papillary muscle, anticipated poor RF contact and deep substrate, upfront PFA was performed. PFA was applied at the earliest activation site, the posteromedial papillary muscle, and further substrate modification of inferior wall and apex was done performed with PFA. A total of 30 applications were required as manipulation of the PFA catheter via the steerable sheath was difficult. There were no PVCs seen immediately after the final ablation. There was no significant reduction in haemoglobin. The ICD lead parameters remained stable post procedure and there were no complications. Cardiac echocardiogram the day after PFA showed an LVEF of 48% with unchanged moderate mitral regurgitation (MR).

## Discussion

This preliminary report highlights the feasibility of PFA in the ablation of intracavitary structures. In all three cases, PVCs originating from intracavity structures (the RV moderator band, RV papillary muscle complex and LV posteromedial papillary muscle) were successfully ablated.

We have made several important findings:

1. PFA delivery to mobile intracavitary structures is feasible with excellent acute success, despite failed prior RF ablations in 2/3 cases.
2. Difficult manoeuvrability of the pentaspline catheter necessitated adjuvant RF ablation in 1/3 cases.
3. PFA delivery to mobile intracavitary structures is safe. The only complication noted was a persistent RBBB with PFA delivery to the moderator band in Case 1. There was no significant reduction in haemoglobin, vasospasm, stroke or deterioration in valvular function.

There are now three published reports describing outcomes of PFA for PVCs (*n* = 21 [14], *n* = 20 [15]  and *n* = 11 [15]). These series, however, included a majority of outflow tract PVCs and all employed focal monopolar ablation using the Centauri system (CardioFocus Inc., MA). Peichl et al. [14] described three patients with papillary muscle arrhythmia (one scar related) and though success rates are not available for this group specifically, at least one is described to have late recurrence at 3 months. In their case series, Della Rocca et al. [15] described five patients (25%) with intracavitary PVCs (two moderator band, two posteromedial papillary muscle, one RV papillary muscle) of which 4/5 had long-term success (recurrence from RV papillary muscle). Four out of five of these cases had irritative ventricular firing, a phenomenon which we did not observe (possibly due to our alternative bipolar PFA system or possibly by chance). Indeed the lack of ventricular ectopy during or after lesion delivery in our series is a counterpoint to ectopy induced during radiofrequency ablation which itself can limit lesion duration. Similar to our Case 1, both patients in this series developed RBBB after ablation of the moderator band (one transient) [15]. Finally in the most recent series by Ruwald et al. [16], the only moderator band case and one of two papillary muscle cases had long-term recurrence. PFA for one of the papillary muscle cases resulted in a minor stroke with remission of symptoms.

It is difficult to compare the success in our series with a pentaspline bipolar ablation catheter against these few cases due to low numbers and different substrate (2/3 patients in our series had PVC-induced VF, 1/3 significant ventricular scar). However, it is conceivable that the catheter shape and large footprint aided catheter contact and lesion delivery and informed differing success rates. We note two recent case reports demonstrating success with pentaspline PFA delivery to the papillary muscles [17, 18]  further supporting our strategy in these difficult to ablate structures.

Important pre-clinical studies have established the ability for PFA to delivery good quality lesions in the ventricle. No significant differences were seen in lesion depth between the focal and basket Farapulse catheters during bipolar PFA in both healthy and infarcted myocardium, though the larger footprint catheter invariably led to increased lesion width [9]. More recently, Nies et al. [11] delivered lesions to intracavitary structures including the papillary muscles and moderator band with ICE guidance using a large footprint monopolar lattice tipped catheter, crucially with ICE, fluoroscopy and electroanatomic mapping guidance. Papillary muscle lesions with good catheter contact had greater lesion dimensions (18.3 mm × 15.3 mm × 5.8 mm deep), whilst lesions with intermittent contact had similar length and width but significantly less depth (3.9 mm, *p* = 0.014).

It is important to note that, as with other studies, the manipulation of such a large, non-deflectable over the wire catheter in ventricle was difficult [19]. This difficulty may be worse within non-dilated ventricles, particularly in the presence of valvular chordae. Furthermore, this limited manoeuvrability can make contact with the ventricular surface difficult. We noted limited manoeuvrability, as reflected in Case 2 and also in Case 3. To address this, our cases were guided by multimodality imaging including 3D mapping, ICE and fluoroscopy, which may help to overcome such difficulty. Despite poor manoeuvrability, we successfully eliminated PVCs from the moderator band in Case 2, possibly due to transmural lesion formed by PFA. Alternatively, the adjuvant effect of RFA may have helped. Further, as shown in Figs. 1, 2 and 3, careful delineation of the target structure with ICE was integral to lesion delivery and could in a large way explain the success of these procedures 3. Others have reported on the manoeuvrability of this system in the ventricle. Importantly, Lozano-Granero et al. [19], were able to target ventricular arrhythmia from a basal LV aneurysm, mid-inferolateral LV and basal inferolateral RV, whilst others have been similarly able to target LV papillary muscle structures [17, 18]. This suggests that this system can afford enough contact for sufficient ablation of arrhythmia at certain sites though certainly alternate sheathes or a deflectable catheter itself may offer promise in addressing this challenge. Another solution that may offer more reach and manoeuvrability for the FaraWave catheter in the ventricle, which was not trialled in this cohort, is the use of an alternative 13 French steerable sheath such as an Agilis NxT Steerable Dual‑Reach sheath (Abbott, IL, USA) where a large curl might facilitate more contact of the PFA catheter with the ventricular endocardium.

Our study did not find significant safety concerns with delivery of PFA to intracavitary structures in the ventricle. Firstly, despite repeated applications with at times poor catheter contact, we report no significant reduction in haemoglobin or significant microbubble emboli. Secondly, although ventricular arrhythmias induced during pre-clinical studies could be attributed to the arrhythmogenicity of the swine model 9, irritative ventricular firing 15 and even initiation of ventricular tachycardia [20] has been noted in human reports. We did not encounter any VF or ectopy during PFA in ventricle and the incidence remained to be studied and determined. Thirdly, in our series, a range of 8–30 PFA applications (with repetitive applications in the same site) were delivered considering the possibility of intermittent contact and swine studies showing repetition improves lesion dimensions [21]. A safety concern could be myocardial stunning by PFA, which may theoretically lead to transient depression of LVEF and even heart failure compensation or hemodynamic instability. This was not observed in our cases; in fact, for our patient in Case 3, echocardiography the day after ablation (and delivery of 30 PFA lesions) saw improvement in EF post ablation (LVEF 41 to 48%). Fourth, ICD lead parameters were not affected by PFA despite proximity of lead to apical ablation site as shown in Cases 1 and 3. We note that the effect of PFA in ventricular ablation on ICD leads is still unclear and potential interactions with ICD leads should not be neglected. Finally, we did note a single incidence of RBBB following ablation of the moderator band which has been described in other studies [15]. Longer-term follow-up is required to understand the effects on the RV of transmural lesion creation in the moderator band.

### Limitations

This study has limitations worth noting. This is a small case series and therefore it is not possible to make treatment comparisons to RF, cryoenergy and alternative PFA delivery systems. We note the relative paucity of cases involving these structures in most ablation cohorts (2–5%). However, these observational results may be of value as a proof-of-concept report describing a strategy to ablate such arrhythmias using PFA guided by ICE such as to inspire further research in this field. Importantly, cryoablation offers promise in treating these structures and this was not offered to any patients in this series. As our cases are limited to acute success and short-term outcomes, further studies are needed to assess long-term outcomes and further delineate the role of PFA in ventricular ablation of intracavitary structures. We did not measure markers of haemolysis such as lactate dehydrogenase, free plasma haemoglobin and haptoglobin and hence subclinical haemolysis may have occurred in these cases which were not detected. Further, variation in voltage delivery, waveform, packet duration, packet number, delivery, catheter configuration and monopolar vs bipolar lesion delivery can all affect procedural success and complications [22]. Determining optimal configurations for lesion formation in these structures therefore require more pre-clinical and clinical validation.

## Conclusion

In conclusion, this case series, though small, provides a proof of concept and novel strategy to ablate difficult to target arrhythmias from intracavitary structures. Further pre-clinical and clinical validation studies are required to establish the role of PFA to treat ventricular arrhythmias.

## Declarations

SK has received research grants from Abbott Medical and Biotronik; speaker’s honoraria from Biosense Webster, Abbott Medical, Biotronik, Medtronic and Sanofi Aventis. All other authors have reported no other relationships relevant to the contents of this paper to disclose.
